# Utilization of immediate postpartum intrauterine contraceptive device and associated factors among mothers who gave birth at selected hospitals in west Gojjam zone, Ethiopia, multi-level facility-based study, 2019

**DOI:** 10.1016/j.heliyon.2021.e06034

**Published:** 2021-01-21

**Authors:** Abenezer Melkie, Dagne Addisu, Maru Mekie, Enyew Dagnew

**Affiliations:** Debre Tabor University, College of Health Sciences, Midwifery Department, Ethiopia

**Keywords:** Immediate, Intrauterine device, Utilization, Ethiopia

## Abstract

**Background:**

Intrauterine contraceptive device (IUCD) prevents unwanted pregnancy. An immediate postpartum period is a good opportunity to place IUCD for women who want to delay pregnancy. However, in Sub-Saharan Africa mainly in Ethiopia, this procedure is not widely used. This study aimed to determine the utilization and factors associated with an immediate postpartum intrauterine contraceptive device (IPPIUCD).

**Methods:**

Multi-level facility-based cross-sectional study was conducted from Januarey12 to March 12/2019GC on 423 women who delivered at selected hospitals of the west Gojjam zone. A systematic random sampling technique was applied to select study participants. Proportional allocations of samples were done based on the delivery caseload of each hospital. Data were entered in Epi info version 7.1 software and exported to SPSS version 23 for editing, cleaning, and analysis. Bivariable and multivariable logistic regression analysis were performed to determine factors associated with the utilization of IPPIUCD.

**Result:**

The utilization of IPPIUCD was 4.02 % (95% CI: 1.65, 5.24). The following factors were significantly associated with IPPIUCD utilization; Being age 35–49 (AOR: 2.98; 1.31, 4.68), College and above education (5.01; 2.21, 7.90), Being counseled about IPPIUD (2.76: 1.79, 7.58), and needing of birth spacing >36 months (2.01: 1.52, 10.12).

**Conclusion:**

The utilization of IPPIUCD was low in selected hospitals of west Gojjam zone. According to this finding; age between 35–49 years, having college and above education, being counseled about IPPIUCD, and needing above 36 months birth spacing were significant factors for utilization of IPPIUCD. Encouraging women's education and informing health professionals of the importance of IPPIUCD may enhance IPPIUCD utilization. This finding may be useful in both reproductive health promotion at an individual level and policy-making regarding this issue.

## 1 Introduction

Intrauterine contraceptive devices (IUCD) is modern contraceptive devices placed in the womb to prevent unwanted pregnancy [1]. The history of IUCD was beginning one hundred years back, even though it has low utilization across the world mainly in Sub-Saharan countries including Ethiopia [1].

Across the world, 10.7 million women have died in the last 25 years between 1990 and 2015 due to maternal causes [2, 3]. Nearly all the global burden of maternal death resides in developing countries. The maternal mortality ratio at the global level is 239 per 100,000 live births. The majority of these maternal deaths occurred in Sub-Saharan Africa [4, 5, 6]. The burden of maternal mortality and morbidity is a growing concern for developing countries, especially in Sub-Saharan Africa due to high rate of unplanned pregnancy and abortion [7, 8, 9]. According to the Ethiopian demographic and health survey(EDHS) 2016, the maternal mortality ratio was 412 deaths per 100,000 live births [10]. Improving contraceptive utilization reduces maternal morbidity and mortality in Sub-Saharan countries [11, 12]. Utilization of long term contraceptive methods reduces maternal deaths by 40 %, infant mortality by 10 %, and childhood mortality by 21 % [11, 13].

In Ethiopia utilization of IUCD was low as compared to other family planning methods [14]. EDHS 2016 showed that <1% of modern family planning users use an immediate postpartum intrauterine contraceptive device (IPPIUCD) [7]. A study done in south nation nationality people of the region showed that the utilization of IPPIUCD was 21.6 %, while 38% of the respondents were interested in using IPPUCD [15].

Improving the use of IPPIUCD can prevent unplanned pregnancy, induced abortion, and maternal death [1, 14]. However, utilization of IPPIUCD is not well-practiced in Ethiopia mainly in the study area due to different reasons. Hence, this study aimed to determine utilization and associated factors of IPPIUCD at selected hospitals of West Gojjam zone. The finding of this study enables policy makers to design appropriate evidence based strategies for better utilization of IPPIUCD.

## 2 Methods and materials

### 2.1 Study area and period

A facility-based cross-sectional study was applied from Januarey12 to March 12/2019 GC at selected in West Gojjam Zone. West Gojjam Zone has seven hospitals. Finote Selam town is the center of the West Gojjam Zone. This town is 332 km away from the capital city of Ethiopia, Addis Abeba.

### 2.2 Source and study population

Source population: Women who gave birth at selected hospitals of west Gojjam zone.

Study population: women who gave birth at selected hospitals of west Gojjam zone during the study period.

### 2.3 Sample size and sampling technique

Sample size was calculated by using a single population proportion formula. The following assumption were used to get the sample size; A 95% confidence level, P-50% proportion, 5% margin of error, and 10% none response rate. Accordingly, the final sample size was 423. Simple random sampling technique was applied to select hospitals. From seven hospitals, four hospitals (Finote Selam, Enjibara, Durbetie, and Danegla) were selected by lottery method. Proportional allocation of samples was done for each hospital based on the number of women who gave birth. Finally, systematic sampling technique was used to select each study participants from selected hospitals.

### 2.4 Data collection technique and tools

The data was collected by using both chart review and paint interview. A structured and pretest questionnaire was used for data collection purposes. Training was given for data collectors and supervisors for two days about the significance of the study, objectives of the study, how to get information from respondents, and how to fill the data on a structured questionnaire. Filled questioners were checked daily by supervisors and investigators for completeness and consistency. Questionnaires were adapted from these articles [15, 16, 17]**(Additional file-1).**

### 2.5 Statistical analysis

Data were entered in Epi info version 7.1 software and exported to SPSS version 23 for editing, cleaning, and analysis. Descriptive statistics was performed to describe the characteristics of the study participants by using percentages and tables. Bivariable and multivariable logistic regression analyses were performed to identify factors associated with the utilization of IPPIUCD. Variables with p-value of <0.2 in the Bivariable analysis were entered into the multivariable model to identify factors associated with IPPIUCD utilization by controlling possible confounding factors at a 95% confidence level. Adjusted odds ratio (AOR) was used to identify factors associated with IPPIUCD utilization. A p-value of <0.05 was used to identify stastically significant variables in multivariable analysis.

### 2.6 Ethical considerations

An ethical clearance letter was obtained from the research ethics committee of Debre Tabor University, College of health sciences. Written consent was obtained from each study participant before the start of data collection. Confidentiality and anonymity of the record were ensured throughout the execution of the study by using codes than the name of the participants.

### 2.7 Variable measurement

#### 2.7.1 Acceptance of IUCD

Woman's verbal consent to use IUCD within 10 min–48 h of delivery of placenta after they counseled about PPIUCD [16].

#### 2.7.2 Utilization of IPPIUCD

Women who had accepted IPPIUCD as the method of family planning and had actual IPPIUCD within 48 h of delivery [15].

#### 2.7.3 Knowledge

The fact that respondents know about IPPIUCD as a method of birth spacing and its benefits. Knowledge was measured by calculating the mean score of 7 items and categorized as having good knowledge (if the participant answered greater than the mean score of knowledgeable questions) or not having good knowledge (if the participant scored less than the mean score of knowledgeable questions) [16].

#### 2.7.4 Attitude

The attitude was measured by calculating the mean score of 5 items and categorized as having a good attitude (if the participant answered greater than the mean score of attitudinal questions) or not having a good attitude (if the participant scored less than the mean score of attitudinal questions) [16].

#### 2.7.5 Counselled about IPPIUCD

If the midwives or other maternity service provider told at least either of benefit, side effect, duration of IPPIUCD during Antenatal care, labor and within 48 h of delivery [15].

## 3 Results

### 3.1 Socio-demographic characteristics

A total of 423 respondents were interviewed with response rate of 100%. The mean ages of the study participants were 29.5 years with a standard deviation of ±4.263. More than seventy percent, 203(70.1%) of the study participants were found in the age range of 25–35 years. The majority of respondents, 379(89.60%) were married (Table 1).

**Table 1:** Socio-demographic characteristics of study participants at selected hospitals in west Gojjam zone, Ethiopia, Multi-level facility-based study, 2019.

Variables and their category	Frequency	Percent
**Age of the mother**		
15–24	102	24.12%
25–34	212	50.12%
35–49	109	25.76%
**Ethnicity**		
Amhara	403	95.27%
Others	20	4.73%
**Religion**		
Orthodox Christian	341	80.61
Muslim	64	15.13%
Others	18	4.26%
**Marital status**		
Married	379	89.60%
Single	19	4.49%
Divorced	18	4.23%
Widowed	7	1.68%
**Education of the mother**		
Unable to read and write	34	8.00%
Able to read and write	91	21.50%
Primary(1–8 Grade)	87	20.80%
Secondary(9–10 Grade)	79	18.67%
Preparatory(11–12 Grade)	68	16.07%
College and above	64	14.84%
**Education of the father**		
Unable to read and write	30	7.09%
Able to read and write	110	26.00%
Primary(1–8 Grade)	76	17.97%
Secondary(9–10 Grade)	68	16.08%
Preparatory(11–12 Grade)	59	13.95%
College and above	80	18.91%
**Distance from the hospital**		
≤3km	150	35.46%
>3km	273	64.54%
**Occupation**		
Governmental employee	132	31.20%
Daily laborer	43	10.17%
Housewife	122	28.84%
Student	29	6.86
Merchant	97	22.93%
**Household income**		
<600	84	19.86%
600–3000	138	32.62%
>3000	201	47.52%

### 3.2 Reproductive, attitude, and knowledge related characteristics

One hundred ninety-eight (46.8%) respondents had a desire to space births for 2–3 years. Nearly two-third, 279 (65.96%) of the respondents discussed methods of family planning with their partner. Almost half, 212(50.11%) of the respondents had 3-4 number of pregnancies (Table 2).

**Table 2:** Reproductive, attitude, and knowledge related characteristics of study participants at selected hospitals in west Gojjam zone, Ethiopia, Multi-level facility-based study, 2019.

Reproductive characteristics	Frequency	Percent (%)
**Number of pregnancy**	N = 423	100%
1–2	212	50.11%
3–4	118	27.89%
5 and above	93	22%
**Number of children**	N = 423	100%
No child	16	3.78%
1-2 children	205	48.46%
3-4 children	112	26.48%
5 and above	90	21.28%
**Current birth planned**	N = 423	100%
Yes	311	73.52%
No	112	26.48%
**Use of family planning before the recent pregnancy**	**N = 423**	**100%**
Yes	269	63.60%
No	154	34.40%
**Plan to have another child in the future**	N = 423	100%
Less than 24 months	86	20.33%
24–36 months	198	46.80%
Above 36 months	118	27.87%
No desire to have children	21	5%
**Ever heard modern FP method**	N = 423	100%
Yes	398	94.09%
No	25	5.91%
**Knowledge on FP**	N = 423	100%
Yes	186	43.97%
No	237	56.03%
**Attitude on FP**	N = 423	100%
Having Good attitude	234	55.32%
Not having a good attitude	189	44.68%
**ANC follow up**	N = 423	100
Yes	254	60.05%
No	169	39.95%
**If yes for the above question frequency of ANC follow up**	N = 423	100
1-2 times	223	52.72%
3-4 times	152	35.93%
5 and above	48	11.35%
**Mood of delivery**	N = 380	100
Spontaneous vaginal delivery	241	56.97%
Instrumental delivery	121	28.60%
Cesarean section	61	14.43%
**Do you discuss with your partner family planning methods?**	N = 423	100
Yes	279	65.96%
No	144	34.04%
**Who decides/will decide on the number of children you want to have?**	N = 423	100
Husband	128	30.26%
Wife	56	13.24%
Both husband and wife	239	56.50%
**Have you got family planning methods now?**	N = 423	100
Yes	70	16.55%
No	353	83.45%
**If your answer is yes which method you used?**	N = 70	100
Jaddelle	10	14.29%
Progesterone only pills(POP)	11	15.71%
Implanon	24	34.28%
IUCD	17	24.28%
Tubal ligation	8	11.44%
**Have you get counseling about IPPIUCD**	N = 423	100%
1.Yes	278	65.72%
2.No	145	34.28%

### 3.3 Utilization of IPPIUCD

The utilization of IPPIUCD among mothers delivered in the selected hospitals of the west Gojjam zone was 4.02 %(95% CI: 1.65, 5.24).

### 3.4 The reason not to use an immediate postpartum intrauterine contraceptive device

**Figure 1:**
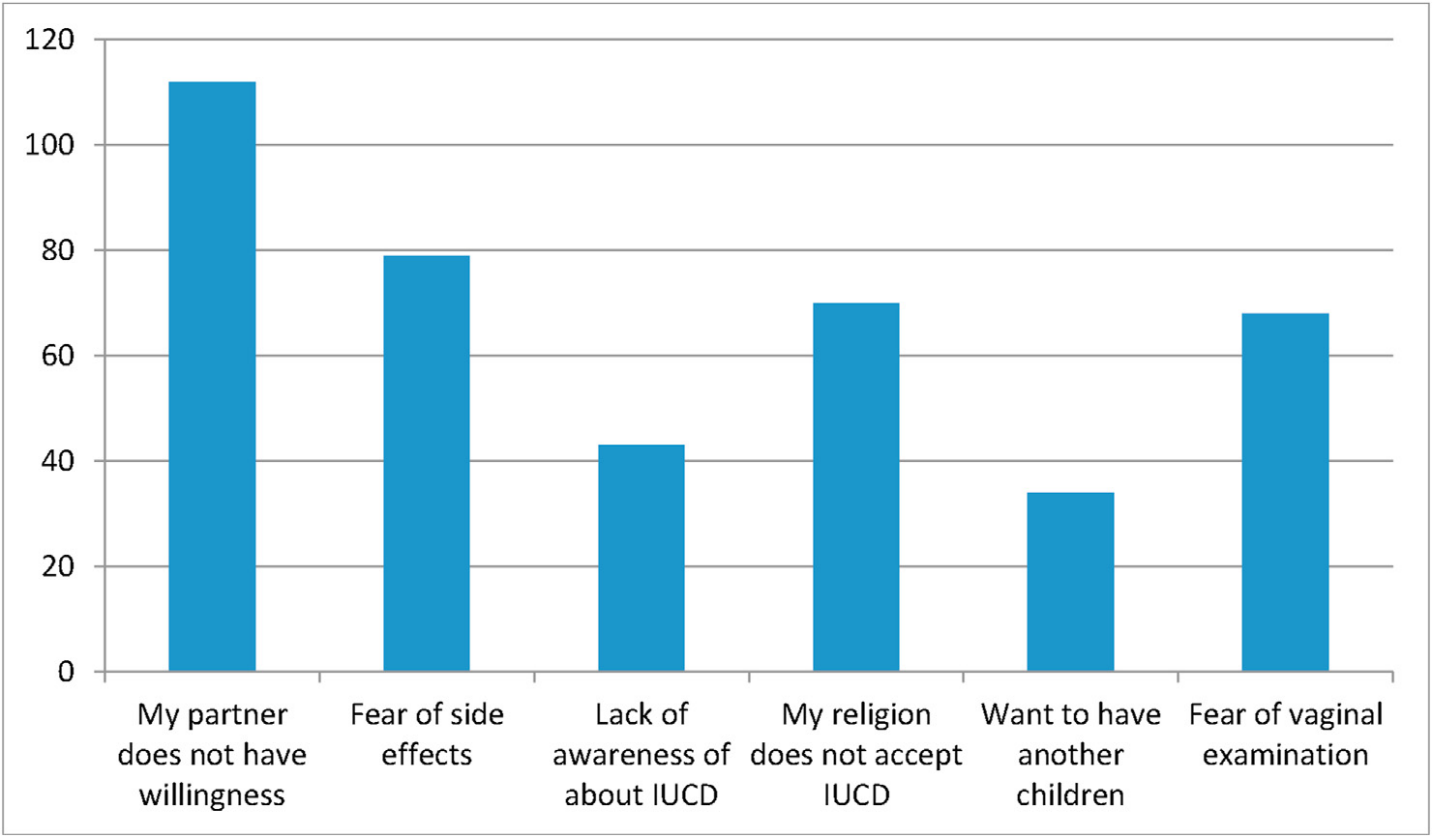
Reason not to use immediate postpartum intrauterine contraceptive device among mothers who gave birth in the selected hospitals of the west Gojjam zone.

The most reason mentioned by participants why they did not use IPPIUCD was couple refusal, 112(55.2%) followed by fear of side effects, 79(27.3%) (Figure 1).

### 3.5 Factors associated with utilization of IPPIUCD

The odds of using IPPIUCD were found to be 2.98 times higher among women in the age group of 34–49 years compared to women who were found in the age group of 15–24 years[AOR = 2.98, 95% CI:(1.31–4.68)]. Those women who had attended college and above level of education were 5.01 times more likely to use IPPIUCD than their counterparts [AOR = 5.01, 95%CI:(2.21–7.90)]. Women who were counseled about IPPIUCD were 2.76 times more likely to use IPPIUCD as compared to those who were not counseled [AOR = 2.76, 95% CI:(1.79–7.58)]. The odds of using IPPIUCD were 2.01 times higher among women who wanted to space childbearing for more than 36 months compared to those who need to space childbearing for less than 24 months [AOR = 2.01,95%CI:(1.52–10.12)] (Table 3).

**Table 3:** Bivariable and Multivariable analysis of independent variables and utilization of IPPIUCD among mothers who gave birth at selected hospitals in west Gojjam zone, Ethiopia, Multi-level facility-based study, 2019.

Variables	Utilization of IPPIUCD		COR(95%CI)	AOR(95%CI)
	Yes	No		
**Age**				
15–24	1	101	1	1
25–34	5	197	2.25(1.05,8.90)	1.23(0.90,5.67)
35–49	11	91	4.34(3.02,8.78)	2.98(1.31,4.68)∗
**Educational status**				
Unable to read and write	1	33	1	1
Able to read and write	3	88	2.03(0.89, 3.2)	1.03(0.78,2.3)
Primary school	2	85	2.05(0.98,3.76)	1.89(0.78,2.98)
Secondary school	1	78	1.67(1.2,7.89)	1.34(0.99,4.67)
Preparatory school	2	66	1.79(1.67,8.12	1.64(0.96,3.78)
College and above	8	56	2.56(2.32,11.32)	5.01(2.21, 7.90)∗
**Have counseling from health professionals on IPPIUCD**				
Yes	11	139	3.23(2.75,12.65)	2.76(1.79, 7.58)∗
No	6	267	1	1
**Birth spacing**				
less than 24 months	1	85	1	1
24–36 months	3	195	3.13(2.15, 8.99)	1.06(0.99,5.34)
above 36 months	12	106	4.02(3.67,12.90)	2.01(1.52–10.12)∗
No desire to have a child	1	20	1.87(1.02,6.04)	1.23(0.58,2.43)

Key: COR: Crude Odds Ratio; AOR: Adjusted Odds Ratio; ∗ significant at a multivariable model.

## 4 Discussion

This study aimed to assess the utilization of IPPIUCD and associated factors among women who gave birth at selected hospitals in the West Gojjam Zone. This study showed that the overall utilization of IPPIUCD among women who gave birth in selected hospitals of the west Gojjam zone was 4.02 %(95% CI: 1.65, 5.24). The finding of this study was found to be low as compared to the studies done in Sidamo Zone and Addis Ababa [15, 18]. The possible reason could be the sample size difference and the difference in the level of awareness of the respondents. The awareness of the women and accessibility of IUCD might be high in Addis Ababa as compared to this study setting. However, our finding was higher than the EDHS 2016 report [10]. This possible explanation could be this study was done in some selected hospitals but EDHS is a large study which was done at the country level.

This study showed that age of women was one of the significant predictors for utilization of IPPIUCD. Mothers in the age group 35–49 years were 2.98 times more likely to use IPPIUCD as compared to mothers in the age group 15–24 [AOR = 2.98, 95% CI:(1.31,4.68)]. This finding is consistent with a study conducted in the Mekelle and Bahir Dar city [14, 17]. The possible explanation could be as age increases couples might have sufficient numbers of families so they want to use long-acting family planning methods like IUCD and others to space or limit the number of their offspring. Besides this, as age increases their exposure to maternal and child health (MCH) services increase so that they might get information and counseling about IPPIUCD at their visit from health professionals.

Educational status was the other significant factor for the utilization of IPPIUCD, those women who had college and above education were 5.01 times higher to use IPPIUCD than to their counterparts [AOR = 5.01, 95%CI:(2.21, 7.90)]. This might be because those women who were educated might have a good attitude, awareness, and knowledge about IPPIUCD.Besides this, they might understand the influence of having large family members on climate change, economic burden, and the educational opportunity of the citizens. Increasing women's educational status is the main strategy to improve the low uptake of IPPIUCD.

This study revealed that participants who had got counseling about IUCD were 2.76 times more likely to be used IUCD as compared to their counterparts [AOR = 2.76, 95% CI:(1.79–7.58)]. Our study is in line with the studies done in the Amhara region [17, 19]. Counseling might decrease misperception and negative attitude about IPPIUCD. Provide counseling for postpartum mothers is a key approach to improve the low utilization of IPPIUCD.

Finally, birth spacing was significantly associated with IPPIUCD utilization. Those women who need above 36 months birth spacing were 2.01 times higher to use IPPIUCD as compared to those who need less than 24 months birth spacing [AOR = 2.01,95%CI:(1.52–10.12)]. This finding was not supported by other studies. The possible explanation could be when women need a long stay to become pregnant they might wish for long-acting family planning methods such as IPPIUCD.

## 5 Conclusion

The utilization of IPPIUCD was low in selected hospitals of the west Gojjam zone. Age group 35–49, having college and above education, being counseled about IPPIUCD, and needing above 36 months birth spacing were significant factors for utilization of IPPIUCD. Encouraging women's education and informing health professionals about the importance of IPPIUCD may enhance IPPIUCD utilization. Besides this, emphasis should be given to IPPIUCD counseling immediately after delivery.

## Declarations

### Author contribution statement

A. Melkie: Conceived and designed the experiments; Performed the experiments; Wrote the paper.

D. Addisu: Performed the experiments; Analyzed and interpreted the data; Wrote the paper.

M. Mekie: Analyzed and interpreted the data; Contributed reagents, materials, analysis tools or data; Wrote the paper.

E. Dagnew: Conceived and designed the experiments; Performed the experiments; Analyzed and interpreted the data.

### Funding statement

This work was supported by Debre Tabor University, College of Medicine and Health Sciences.

### Data availability statement

Data will be made available on request.

### Declaration of interests statement

The authors declare no conflict of interest.

### Additional information

No additional information is available for this paper.
