# Supplementary material for: Utilization of immediate postpartum intrauterine contraceptive device and associated factors among mothers who gave birth at selected hospitals in west Gojjam zone, Ethiopia, multi-level facility-based study, 2019
Source: Heliyon. 2021 Jan 21;7(1):e06034. doi: 10.1016/j.heliyon.2021.e06034 (PMC7820927; doi:10.1016/j.heliyon.2021.e06034)
Supplement: Additional_file-1 [file mmc1.docx]

**Additional file-1**

**Questionnaire for assessing utilization of immediate postpartum intrauterine contraceptive device and associated factors among mothers who gave birth at selected hospitals in west Gojjam zone, Ethiopia, Multi-level facility based study, 2019**

**Participant code__________ Date______________**

| **S.No** | **Part I: Socio-demographic characteristics of the study participants** | |
| --- | --- | --- |
| 001  . | Age | _____(completed years) |
| 002 | Ethnicity | 1.Amhara  2.Oromo  3.Tigre  4.Gurage  5.Other(specify)____ |
| 003 | Religion | 1. Orthodox 2. Muslim 3. Protestant 4. Catholic 5. Other(specify)_____ |
| 004 | Marital status | 1.Married  2. Single  3. Divorced  4. Widowed |
| 005 | Education of the mother | 1. Unable to read and write 2. Able to read and write 3. Primary( 1-8 Grade) 4. Secondary(9-10 Grade) 5. Preparatory( 11-12 Grade) 6. College and above |
| 006 | Education of the father | 1. Unable to read and write 2. Able to read and write 3. Primary( 1-8 Grade) 4. Secondary(9-10 Grade) 5. Preparatory( 11-12 Grade) 6. College and above |
| 007 | Distance from the hospital | 1.≤3km  2.>3km |
| 008 | Occupation | 1.Governmental employee  2.Daily laborer  3.Housewife  4.Student  5.Merchant |
| 009 | Household income(Ethiopian birr) | <600 birr  600-3000 birr  >3000 birr |
| ***Part* II: Reproductive characteristics of the study participants** | | |
| 011 | How many times ever been pregnancy? | 1.______________ |
| 012 | How many children do you have? | 1._____________ |
| 013 | Is the current pregnancy planned? | 1.Yes  2.No |
| 014  015 | Did you use family planning methods before the recent pregnancy?  When will be your next pregnancy ? | 1.Yes  2.No  1.Less than 24 months  2.24-36 months  3.Above 36 months  4.No desire to have children |
| 016 | Have you ever heard modern FP method? | 1.Yes  2.No |
| 017 | Did you have ANC follow up? | 1.Yes  2.No |
| 018 | If yes for the above question frequency of ANC follow up | 1. one  2. Two  3.Three  4.Four  5.five and above |
| 019 | Mode of delivery | 1. Spontaneous vaginal delivery 2. Instrumental delivery 3. Cesarean section |
| 020 | Do you discuss with your partner about family planning methods? | 1.Yes  2. No |
| 021 | Who decides/will decide on the number of children you want to have? | 1.Hasband  2.Wife  3.Both husband and wife |
| 022 | Have you got family planning methods now? | 1.Yes  2.No |
| 023 | If your answer for the above question is “yes” which method you used? | 1.Jaddelle  2. Progesterone only pills(POP)  3. Implanon  4.IUCD  5. Tubal ligation  6.Other(specify)________________ |
| 024 | Have get counseling about IPPIUCD? | 1.Yes  2.No |
| 025 | Do you get IPPIUCD? | 1.Yes  2.No |
| **Part III: Knowledge related characteristics of the study participants** | | |
| 026 | Have you ever heard about IPPIUCD? | 1.Yes  2.No |
| 027 | IPPIUCD can be used for HIV positive patients. | 1.Yes  2.No |
| 028 | Does IPPIUD prevent pregnancies 10-12 years? | 1.Yes  2.No |
| 029 | IPPIUD is inserted free of charge in Ethiopia. | 1.Yes  2.No |
| 030 | Does IPPIUCD immediately reversible (become pregnant quickly when removed). | 1.Yes  2..No |
| 031 | Do breast feeding mothers can use IPPIUCD? | 1.Yes  2.No |
| 032 | Can you remove IPPIUCD at any time you wish? | 1.Yes  2.No |
| **Part IV: Attitude related characteristics of the study participants** | | |
| 033 | Insertion of IPPIUCD inside the uterus does not have effect on clients’ privacy. | 1.Yes  2. No  . |
| 034 | Using IPPIUCD does not restrict normal activities. | 1.Yes  2.No  . |
| 035 | IPPIUCD doesn’t move through the body after insertion. | 1.Yes  2.No |
| 036 | IPPIUCD can harm a woman’s womb. | 1.Yes  2.No  . |
| 037 | IPPIUCD doesn’t affect the sexual desire of your partner. | 1.Yes  2.No |
